# The Influence of Contamination and Different Cleaning Methods and the Effect of Plasma Treatment of CoCr Alloy on Tensile Bond Strength to Composite Resin

**DOI:** 10.3290/j.jad.b5200039

**Published:** 2024-04-11

**Authors:** Tuğba Arslan, Sebastian Wille, Matthias Kern

**Affiliations:** a Dentist, Clinic for Conservative Dentistry and Periodontology, Christian-Albrecht University, Kiel, Germany. Performed the experiments in partial fulfillment for her doctoral thesis, wrote the manuscript.; b Research Scientist, Department of Prosthodontics, Propaedeutics and Dental Materials, School of Dentistry, Christian-Albrecht University, Kiel, Germany. Consulted on and contributed to the statistical elevation, contributed to the experimental design, supervised the experiments, reviewed and edited the manuscript.; c Professor and Chairman, Department of Prosthodontics, Propaedeutics and Dental Materials, School of Dentistry, Christian-Albrecht University, Kiel, Germany. Developed the idea, hypothesis and experimental design, reviewed and edited the manuscript.

**Keywords:** alloy, contamination, cleaning, bonding, plasma treatment

## Abstract

**Purpose:**

To investigate the influence of contamination and different cleaning methods on resin bonding to cobalt-chromium (CoCr) alloy disks.

**Materials and Methods:**

A total of 160 CoCr disks were divided into 3 groups. The first group (N = 64) was air abraded with alumina particles and contaminated with a silicone disclosing agent and saliva; the second group (N = 64) was air abraded but not contaminated; the third group (N = 32) was neither air abraded nor contaminated. The first two groups were divided into 4 subgroups (N = 16) according to the cleaning method: ultrasonic bath in 99% isopropanol, use of a cleaning suspension of zirconium oxide particles, use of a cleaning suspension based on 10-MDP salt, and treatment with atmospheric plasma. The third group was divided into 2 subgroups (N = 16): treatment with atmospheric plasma and no treatment. All CoCr specimens were bonded to plexiglas tubes filled with a bonding resin that contained phosphate monomer. Tensile bond strength (TBS) was examined by tensile testing after 3 and 150 days of water storage plus 37,500 thermal cycles (N = 8).

**Results:**

After contamination, TBS was significantly reduced after 150 days of water storage. Groups without air abrasion showed initially low TBS and debonded spontaneously after 150 days of water storage.

**Conclusion:**

None of the cleaning methods was able to remove saliva and silicone disclosing agent on CoCr-alloy surfaces. Surface activation by plasma treatment has no long-term effect on the bond strength.

Dental alloys continue to be used in many types of restorations in dentistry. These include implant superstructures, metal-ceramic crowns, and resin-bonded metal retainers. Despite the availability of dental implants, metal-ceramic resin-bonded fixed dental prostheses (RBFDPs) and metal resin-bonded attachments (RBAs) for removable dental prostheses are still standard therapies. Depending on the composition of the alloy, various surface treatments are recommended to improve the bond strength between the luting resin and the alloy.^6,7,14,22^ These include air abrasion, tin plating, silica coating, and the application of metal primers.^1,15,25,43^ Resin luting systems that contain phosphate monomers allow adhesive bonding of alloy and ceramic restorations to dentin and enamel.^36,58,59^

Adhesive bonding is a technique-sensitive procedure, characterized in one study by debonding and minor fractures over 5 years.^49^ A prerequisite for durable resin bonding is that the bonding surfaces of the restorations are clean.^42,55^ Clinically, contamination of the bonding surface can result in a significant reduction of the bond strength.^8^ Try-in procedures of dental restorations are required before final insertion. During the try-in procedure, contamination with saliva, blood, or silicone disclosing agents often cannot be avoided.^38,42,55^ As a result, cleaning methods have been tested in several laboratory studies in order to remove the contamination and provide strong resin bonding. Cleaning the contaminated surfaces by rinsing with water, ultrasonically in isopropanol, or a combination of the two has shown a minor or no cleaning effect on zirconia surfaces.^3,42,54,55^ Another method, airborne-particle abrasion with alumina particles, has been reported to effectively remove contaminants from dental alloys, resulting in sufficient bond strengths if it is used in combination with an 10-MDP-containing adhesive or primer.^1,52^

An alternative cleaning method is the use of non-thermal atmospheric plasma.^16,40^ An appropriate cleaning method ideally has no negative side effects. High efficacy in removing contaminants and ensuring a strong resin bond to alloy is desirable. Plasma is a gaseous condition containing electrons, ions, and neutral particles, and is defined as the fourth state of matter.^32,39^ The interaction of plasma with the surface of various materials can induce different effects on the material surface: the removal of organic substances, cross-linking of film-forming groups, ablation, and surface chemical restructuring.^33,48^ Sterilizing surgical instruments, disinfecting carious dentin, and the elimination of dental bacteria are examples for dental applications of non-thermal atmospheric plasma.^12,19,45,47^ In recent years, the idea of using non-thermal atmospheric plasma to improve adhesive bonding of dental restorations has gained scientific interest. Treatment with non-thermal atmospheric plasma increases the surface energy and makes a more hydrophilic surface on materials such as dentin, enamel, composite, and ceramics.^10,17,44^ It has been proven that the adhesive bond between tooth and zirconia and between zirconia and composite is improved by the increased surface energy.^5,51^ In contrast, another study reported that plasma treatment disturbs the bonding between the cobalt-chromium (CoCr) alloy and self-curing acrylic resin, despite the high degree of activity on the surface after treatment with plasma.^23,34^ It has been shown that plasma has a cleaning effect on ceramic surfaces contaminated with saliva and silicone, but it was less effective than alumina-particle air abrasion.^16,40^ To the authors’ best knowledge, the cleaning effect on contaminated CoCr-alloy surfaces has not yet been investigated.

Another approach to remove contamination comprises cleaning agents based on a basic (pH ≥7) suspension of zirconium oxide particles, or those which contain 10-MDP salt as a main component.

Manufacturers claim that after application of the cleaning suspension, water rinsing and air drying effectively removes saliva and silicone contaminants from the surface of restorations. Several studies have shown that treatment with a cleaning agent based on a suspension of zirconium oxide particles does not result in bond strength durability similar to that after conventional treatment of ceramic surfaces. However, bond strengths after cleaning with such a suspension were better than treatment with isopropanol, water rinsing, or phosphoric acid alone.^2,13,31,46^ However, to the authors’ best knowledge, the cleaning effect of suspensions of zirconium oxide particles or those based on 10-MDP salt on CoCr-alloy surfaces after contamination with saliva and silicone and its influence on TBS have not yet been investigated.

For clinical use, the simplification of the cleaning procedure by using a suspension would be advantageous, provided that the surface is cleaned reliably. Furthermore, the use of plasma could provide an enhanced cleaning effect. However, it is also important to clarify whether the negative effect on bond strength from plasma activation^34^ outweighs the benefits of cleaning. Therefore, the aim of this study was to investigate the use of different cleaning methods as possible alternatives to alumina-particle air abrasion of CoCr surfaces contaminated with saliva and silicone. Additionally, the influence of treating CoCr surfaces with air plasma on the tensile bond strength (TBS) of resin bonding was examined.

The first null hypothesis of this study was that the contamination and cleaning methods have no influence on TBS. The second null hypothesis was that treatment of a non-contaminated surface with atmospheric plasma has no effect on TBS.

## Materials and Methods

### Specimen Preparation

In this study, 160 CoCr-alloy disks (Wirobond C, BEGO; Bremen, Germany) with a diameter of 7.5 ± 0.5 mm and a thickness of 3.5 ± 0.5 mm were used. The bonding surfaces of the disks were polished with 600-grit abrasive silicon carbide paper under water rinsing.

The specimens were randomly divided into 3 groups. The first group (N = 64) was airborne-particle abraded with 50-μm alumina particles at 0.25 MPa pressure at a distance of 10 mm. Afterwards, the first group was ultrasonically cleaned in 99% isopropanol for 3 min and dried using a blast of oil-free air.

Then the first group (airborne-particle abraded, “A”) was divided into 4 subgroups (N = 16) according to the cleaning method: ultrasonic bath in 99% isopropanol (A-NC(no contamination)-IP), use of a cleaning suspension of zirconium oxide particles (Ivoclean, Ivoclar; Schaan, Liechtenstein) (A-NC-IV), use of a cleaning suspension based on 10-MDP salt as a main component (Katana Cleaner, Kuraray Noritake; Osaka, Japan) (A-NC-KC), and treatment with plasma (A-NC-P). The cleaning suspension of zirconium oxide particles was applied to the surfaces of the specimens for 20 s, then rinsed with water spray for 20 s, followed by drying with a blast of oil-free air.

Treatment with the cleaning suspension based on 10-MDP salt was identical to that of the suspension of zirconium oxide particles, but with a shorter application time of 10 s.

In this study, atmospheric plasma of a low vacuum non-thermal-plasma chamber (Femto PCCE, diener electronic; Ebhausen, Germany) was used. A high-frequency generator produced an asymmetric, capacitively coupled radio-frequency (rf) discharge of up to 200 W and a frequency of 100 kHz. The duration of the plasma treatment was 5 min and the cleaning process was carried out at a working pressure of 50 Pa.

The second group (N = 64) was alumina-particle air abraded, ultrasonically cleaned in 99% isopropanol, followed by contamination with a silicone-disclosing agent and saliva. The silicone-disclosing agent (Fit Checker Advanced, GC Germany; Bad Homburg, Germany) was mixed according to the manufacturer’s instructions. A thin layer was applied on the subsequent bonding surface of the specimens and placed into a 37°C incubator for one minute. Afterwards, the silicone layer was removed with tweezers. Unstimulated saliva was applied using micro brushes on the surfaces of the specimens for 10 s. After 20 s of absorption, the surfaces were rinsed with water spray for 15 s and dried with a blast of oil-free air. Saliva was collected from one healthy female donor who had refrained from eating and drinking 1.5 h prior to the collection procedure. All experiments were performed using fresh saliva collected on the same occasion. Subsequently, the specimens of the second group were divided into 4 subgroups (N = 16) according to the same cleaning methods as the first group (A-C-IP, A-C-IV, A-C-KC and A-C-P).

The third group (N = 32) was neither air abraded nor contaminated. The specimens of the third group were ultrasonically cleaned in 99% isopropanol for 3 min and dried using a blast of oil-free air. Then they were randomly divided into 2 subgroups (N = 16): one subgroup was treated with air plasma (NA-NC-IP), the other subgroup did not undergo any treatment (NA-NC-P).

**Fig 1:**
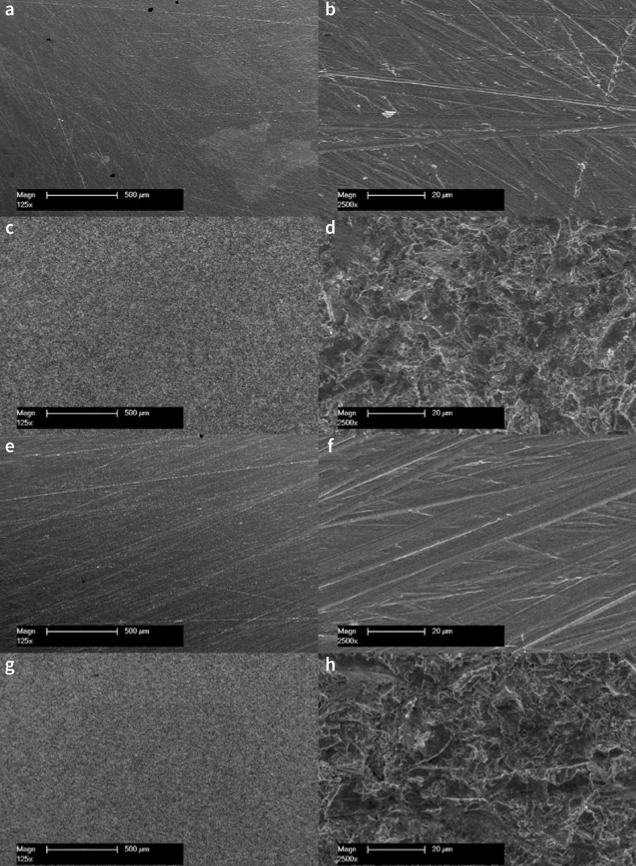
Representative scanning electron micrographs of CoCr surfaces. (a) Air abraded and ultrasonically cleaned in 99% isopropanol (A-NC-IP) (125X magnification); (b) air abraded and ultrasonically cleaned in 99% isopropanol (A-NC-IP) (2500X magnification); (c) ultrasonically cleaned in 99% isopropanol without air abrasion (NA-NC-IP) (125X magnification); (d) ultrasonically cleaned in 99% isopropanol without air abrasion (NA-NC-IP) (2500X magnification); (e) air abraded, ultrasonically cleaned in 99% isopropanol and plasma-treated (A-NC-P) (125X magnification); (f) air abraded, ultrasonically cleaned in 99% isopropanol, and plasma-treated (A-NC-P) (2500X magnification); (g) ultrasonically cleaned in 99% isopropanol and plasma-treated without air abrasion (NA-NC-P) (125X magnification); (h) ultrasonically cleaned in 99% isopropanol and plasma-treated without air abrasion (NA-NC-P) (2500X magnification).

The surfaces of representative specimens of the groups – A-NC-IP, A-NC-P, NA-NC-IP and NA-NC-P – were examined using a scanning electron microscope (SEM, XL 30 CP, Philips; Eindhoven, Netherlands) with an acceleration voltage of 15 keV (Fig 1).

Batch numbers and composition of the materials used are shown in Table 1. The pretreatment, contamination, and cleaning methods of test groups and their subgroups are listed in Table 2.

**Table 1:** List of materials and the corresponding composition and batch number used in this study

Material	Company	Composition*	Batch No.
Wirobond C	Bego; Bremen, Germany	Co 63.3%, Cr 24.8%, W 5.3%, Mo 5.1%, Si 1.0%	4085
GC Fit Checker white advanced, base	GC; Tokyo, Japan	Silicon dioxide, vinyldimethylpolysiloxane, polyether compound, methylhydrogen dimethylpolysiloxane	1907231
GC Fit Checker white advanced, catalyst	GC	Vinyldimethylpolysiloxane, silicon dioxide, titanium dioxide	1907231
Clearfil Core New Bond, base	Kuraray Noritake; Osaka, Japan	Bis-GMA, TEG-DMA, DMA	000089
Clearfil Core New Bond, catalyst	Kuraray Noritake	Barium sulfate, silica-containing resin composite	000089
Panavia V5 Paste opaque	Kuraray Noritake	Bis-GMA, TEG-DMA, hydrophilic aliphatic and hydrophobic aromatic dimethacylates, initiators, accelerators, silanated barium glass filler, silanated fluoroalminosilicate glass filler, colloidal silica bisphenol A, silanated alminium oxide filler, dl-camphorquinone, pigments	1H0008
Liquid Strip	Ivoclar; Schaan, Liechtenstein	Glycerin gel	W11589
Ivoclean	Ivoclar	Polyethylene glycol, sodium hydroxide	X09287
Katana Cleaner	Kuraray Noritake	Triethanolamine, polyethyleneglycol, 10-MDP salt	AG0001
Clearfil Ceramic Primer Plus	Kuraray Noritake	3-methacryloxypropyl trimethoxysilane, 10-MDP, ethanol	9S0030

Bis-GMA: bisphenol-A-diglycidylmethacrylate; DMA: aliphatic dimethacrylate; 10-MDP: 10-methacryloyloxy-decyl dihydrogenphosphate; TEG-DMA: triethyleneglycol dimethacrylate. *Manufacturers’ information.

**Table 2:** Groups and subgroups and their surface treatments

Groups and subgroups (N = 16)	Air abrasion	Contamination	Cleaning
A-NC-IP	Air abrasion	No contamination	Isopropanol
A-NC-IV	Air abrasion	No contamination	Ivoclean
A-NC-KC	Air abrasion	No contamination	Katana Cleaner
A-NC-P	Air abrasion	No contamination	Atmospheric plasma
A-C-IP	Air abrasion	Saliva and Fit Checker	Isopropanol
A-C-IV	Air abrasion	Saliva and Fit Checker	Ivoclean
A-C-KC	Air abrasion	Saliva and Fit Checker	Katana Cleaner
A-C-P	Air abrasion	Saliva and Fit Checker	Atmospheric plasma
NA-NC-IP	No air abrasion	No contamination	Isopropanol
NA-NC-P	No air abrasion	No contamination	Atmospheric plasma

### Tensile Bond Strength Testing

Prefabricated plexiglas tubes with an inner diameter of 3.2 mm (adhesive surface of ca 8.04 mm^2^) and a length of 15.5 mm were filled with self-curing resin composite (Clearfil DC Core New Bond, Kuraray Noritake). After polymerization of the composite for 7 min, the filled tubes were bonded with a dual-curing luting resin (Panavia V5 opaque, Kuraray Noritake) to the CoCr-alloy bonding surface using an alignment apparatus under a load of 750 g.

Before bonding, a phosphate monomer-containing primer (Clearfil Ceramic Primer Plus, Kuraray Noritake) was applied on the bonding surfaces of the CoCr-alloy specimens. After application of the corresponding primer, the surfaces were dried with oil-free air. The alignment apparatus ensured that the tube axis was perpendicular to the bonding surface.^20^ Excess cement was removed with foam pellets. To prevent an oxygen-inhibited polymerization layer, a glycerol gel (Liquid Strip, Ivoclar) was applied around the bonding margins. The margins were light cured from 2 opposite sides using a light-curing unit (radii-cal, SDI; Bayswater, Victoria, Australia) for a total of 40 s. Afterwards the bonded specimens were light polymerized in the curing apparatus (HiLite power, Heraeus Kulzer; Hanau, Germany) for 90 s to ensure complete curing of the resin.

The bonded specimens were randomly divided into 2 subgroups with 8 specimens per subgroup. For artificial aging, these 2 subgroups were stored in 37°C water for 3 days or for 150 days with an additional 37,500 thermal cycles between 5°C and 55°C with a dwell time of 30 s.^42,53^ The tensile bond strength (TBS) was measured after the different storage conditions using a universal testing machine (Zwick Z010/TN2A, Zwick Roell; Ulm, Germany) at a crosshead speed of 2 mm/min with a chain loop alignment that ensured moment-free axial application.^20,29^

Statistical analysis was carried out using SPSS (Version 20, IBM; Armonk, NY, USA). The Shapiro-Wilk test showed that only a few groups were not normally distributed. Homogeneity of variances was examined with Levene’s test. The significance level α was set at 0.05 and Levene’s test showed that there was no homogeneity of variances. Therefore, the statistical analysis was performed with the Kruskal-Wallis test. The Mann-Whitney U-Test was used for pairwise comparison, followed by the Bonferroni-Holm correction.

### Failure Mode

A light microscope (Wild M 420 Heerbrugg, Germany) at a magnification of 25X was used to examine the debonded, fractured interfaces of all CoCr-alloy specimens. The failure modes were classified as adhesive failure at the CoCr-alloy disk surface or cohesive failure in the luting resin composite or the composite-filled tube.

If CoCr was visible, failure was classified as adhesive; if not, it was classified as cohesive. The proportion of cohesive and adhesive failures was determined for each specimen by calculating the areas observed for each type of failure and expressing them as a percentage of the total bonded area. Afterwards, the mean value for each group was determined.

**Fig 2:**
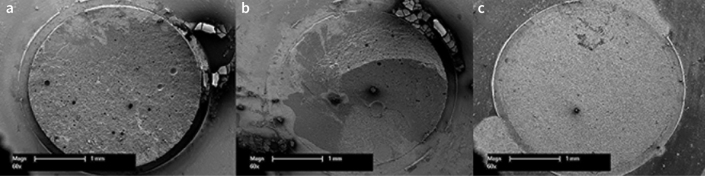
Representative scanning electron micrographs of failure modes (50X magnification). (a) Completely cohesive failure; (b) mixed failure; (c) completely adhesive failure.

Additionally, representative specimens were examined using a scanning electron microscope (SEM, XL 30 CP, Philips) with an acceleration voltage of 15 keV after sputter-coating with a 15-nm gold-alloy conductive layer (Leica EM QSG 100; Wetzlar, Germany) (Fig 2).

## Results

### Tensile Bond Strength

Table 3 shows medians, means, and standard deviations of the TBS of all groups in MPa. Without contamination, there were no statistically significant differences between the different cleaning methods after 3 days and 150 days. Specimens that failed spontaneously during thermal cycling and 150-day water storage were evaluated as having a TBS of 0 MPa, resulting in a median TBS of 0 MPa in groups A-C-IP, A-C-P, NA-NC-IP, and NA-NC-P. Specimens of the other groups that showed no debonding during artificial aging were evaluated by TBS testing. Using Katana Cleaner after contamination (A-C-KC) resulted in statistically significant higher TBS than after using isopropanol cleaning (A-C-IP) or plasma cleaning (A-C-P) after 3 and 150 days of storage. The specimens of all contaminated groups (A-C-IP, A-C-IV, A-C-KC, and A-C-P) showed a significantly lower TBS compared within the non-contaminated groups which were air abraded (A-NC-IP, A-NC-IV, A-NC-KC and A-NC-P). All groups without air abrasion (NA-NC-IP and NA-NC-P) also showed significantly lower TBS compared within the uncontaminated and air-abraded groups (A-NC-IP, A-NC-IV, A-NC-KC, and A-NC-P). The TBS of both groups without air abrasion (NA-NC-IP and NA-NC-P) did not differ significantly. However, the TBS of these two groups (NA-NC-IP and NA-NC-P) decreased significantly during artificial aging over 150 days.

**Table 3:** Medians, means, and standard deviations (SD) in MPa of the tensile bond strength (TBS) of test groups (N = 8)

Cleaning method		Air abrasion				No air abrasion	
		No contamination		Saliva and silicone		No contamination	
		3-day water storage	150-day water storage	3-day water storage	150-day water storage	3-day water storage	150-day water storage
Iso-propanol	Median	45.1^aA^	32.0^aA^	10.2^bA^	0.0^bB^	9.0^aA^	0.0^aB^
	Mean	46.6	32.5	8.8	0.4	10.3	0.0
	SD	8.2	7.6	4.0	0.8	3.5	0.0
Ivoclean	Median	42.3^aA^	32.5^aA^	19.5^abA^	7.3^abA^	---	---
	Mean	43.4	32.7	19.4	8.5		
	SD	8.1	7.1	8.4	7.0		
Katana Cleaner	Median	50.4^aA^	30.3 aB	31.3^aA^	10.8^aB^	---	---
	Mean	51.7	29.5	29.5	12.4		
	SD	6.2	5.1	8.9	3.7		
Plasma	Median	42.5^aA^	29.1^aA^	14.0^bA^	0.0^bB^	17.6^aA^	0.0^aB^
	Mean	41.4	29.4	15.1	1.8	17.5	0.0
	SD	10.7	6.9	3.3	3.4	6.5	0.0

Statistically significantly different groups (p≤0.05) are indicated by different superscript lowercase letters within a column and different uppercase letters within a row, comparing 3 and 150 days storage within the same test group.

### Failure Modes

**Fig 3:**
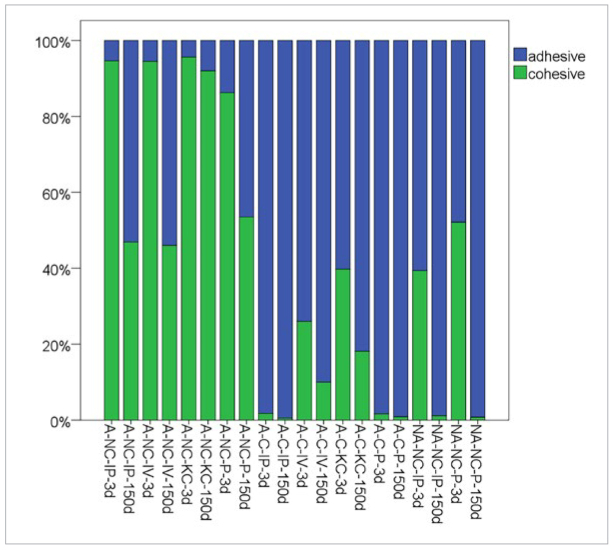
Mean percentages of areas showing adhesive failure at the CoCr surface and cohesive failure in the luting resin composite or in the tube-filling resin composite.

In Fig 3, the percentages of debonded areas corresponding to cohesive and adhesive failure are shown. Groups with a high TBS showed a predominantly cohesive failure. The proportion of cohesive failures decreased for groups with lower TBS. Groups with very low TBS or groups that spontaneously debonded exhibited almost completely adhesive failure. SEM images of debonded specimens exemplify the failure modes.

## Discussion

The first null hypothesis has to be rejected, because there were statistically significant differences in the TBS with and without contamination and also between individual cleaning methods after contamination. The second null hypothesis has to be accepted, because the additional treatment with air plasma had no significant effect on the TBS.

The results of this study confirm previous studies that air abrasion with alumina particles and contamination of CoCr-alloy surfaces influence the adhesion of resin bonding.^11,42,53,60^ To show the long-term influence of contamination on resin bonding, a standardized water storage and thermal cycling procedure was used. Thermal cycling is an effective procedure to simulate aging.^26,27^ Temperature changes and water absorption are important factors which influence the durability of resin bonds. Thermocycling also serves to stress the resin-metal bond due to the large difference in the thermal expansion coefficient between the metal substrate and the composite resin.^26,28^

Since all cleaning methods showed high TBS after 3 and 150 days for uncontaminated air-abraded surfaces, it can be assumed that none of the cleaning methods caused additional contamination of the bonding surface.

Saliva and silicone contamination had a negative impact on the TBS. Previous studies with silicone contamination on ceramic surfaces showed either a significant impact^40,55^ or no significant impact on the TBS.^16^ In this study, where no negative effect of silicone contamination on the TBS was shown, the silicone-covered specimens were put in an incubator at 37°C for 3 min. This was intended to simulate the intraoral temperature during clinical try-in. The incubation time of 3 min might lead to complete polymerization, and thus no residuals were observed on the ceramic surfaces.^16^ The incubation time of 1 min was chosen to investigate the consequences of shortening the indicated curing time and thus incomplete polymerization with regard to the adhesive bond.

Saliva is mainly composed of organic elements, such as proteins, bacteria, enzymatic molecules and food debris in an aqueous solution.^50^ Salivary protein adsorption takes place on tooth surfaces^4^ and on the surfaces of the restorative dental materials.^18,35^ If the alloy surfaces are insufficiently cleaned, saliva is one of the factors which affects TBS negatively.^24,26,37^

One of the problems with contamination with saliva is the fixation of proteins on surfaces caused by the use of alcohol-containing media. Denatured protein residuals are difficult to remove and can further form a biofilm. For medical instrument processing, non-fixing detergents are therefore recommended. Cleaning by isopropanol alone yielded low TBS and insufficient adhesion values for use in dentistry.^9,16,41^ To the authors’ knowledge, there is no comparable study on the cleaning effect of isopropanol alone on contaminated surfaces of CoCr alloys. However, previous studies on contaminated zirconia samples showed that isopropanol was not able to sufficiently remove saliva and silicone disclosing agent residues from the samples.^3,40,42,55^

The bond strength of the specimens treated with the zirconia-particle suspension without contamination (A-NC-IV) was not significantly different from that of the control group (A-NC-IP). In contrast, the bond strength after contamination (A-C-IV) decreased significantly. The zirconia-particle suspension did not sufficiently clean the contaminated CoCr-alloy surfaces, as TBS after 150 days of water storage plus thermocycling was significantly reduced compared to the air-abraded, uncontaminated groups (A-NC-IV). Therefore, the cleaning effect of this suspension alone after contamination is not sufficient.

To the authors’ best knowledge, the cleaning effect of this zirconia suspension on alloy surfaces has not been previously investigated. In other studies, zirconia specimens were cleaned with solution based on basic suspension of zirconia particles after contamination with saliva. After thermocycling, the bond strength decreased significantly compared to the initial bond strength.^21,56,57^

The specimens which were additionally treated with the 10-MDP-salt suspension (A-C-KC) showed significantly higher TBS after 150 days than the group cleaned with isopropanol (A-C-IP). However, TBS decreased significantly after thermocycling and water storage over 150 days compared to the air-abraded, uncontaminated group (A-NC-KC). These results show that the cleaning effect of this suspension is not sufficient. To the authors’ best knowledge, the cleaning effect of this suspension on contaminated CoCr alloy has not been previously determined; however, an earlier study showed that the 10-MDP-salt suspension had a statistically significant cleaning effect on saliva-contaminated zirconia surfaces.^46^ This effect is not confirmed by the results of the present study transferred to CoCr surfaces. As the TBS was not clinically sufficient after contamination and cleaning with the 10-MDP-salt suspension after 150 days of water storage, this suspension does not seem adequate to remove contamination with saliva and silicone from CoCr alloy.

Plasma generates reactive species and functional groups, and breaks chemical bonds such as C-H and C-C. This mainly chemical process allows gentle cleaning without degrading surfaces, which is an advantage especially when dealing with ceramics.^11^ However, a previous study demonstrated that plasma cleaning alone was not powerful enough to remove silicone remnants on contaminated zirconia surfaces.^40^ The insufficient cleaning effect of plasma might be due to incompletely polymerized silicone remnants. This would imply that silicone must be completely polymerized in order for the plasma to sufficiently clean the contaminated surfaces. Other studies showed the effectiveness of a combined chemo-mechanical method using an ultrasonic bath filled with 99% isopropanol solution followed by plasma treatment for cleaning saliva-contaminated zirconia surfaces.^40^ Analogous to the cleaning effects of plasma on zirconia surfaces contaminated with saliva, these effects of plasma can possibly also be transferred to CoCr surfaces. However, this should be investigated in a separate study.

The bond strength of adhesive resin to titanium was higher after exposure to air plasma than the bond strength to untreated titanium.^30^ In contrast, a previous study in which CoCr specimens were treated with plasma showed that plasma treatment not only did not improve adhesion of a self-curing adhesive resin to CoCr alloy but did in fact disturb resin bonding to CoCr alloy.^34^ In the current study, the bond strength in plasma-treated, uncontaminated CoCr specimens did not improve compared to the other uncontaminated groups without plasma treatment. However, as opposed to the findings by Maruo et al,^34^ plasma treatment had no statistically significant negative effect on the TBS. Comparing the images of group A-NC-IP (Figs 3a and 3b) with the images of group A-NC-P (Figs 3e and 3f), no differences are visible on the surfaces.

In the present study, the TBS of the plasma-treated groups without air abrasion (NA-NC-P) was low and is considered clinically unacceptable. Comparing the images of the NA-NC-IP group (Figs 3c and 3d) with the images of the NA-NC-P group (Figs 3g and 3h), it can be seen that the treatment with air plasma has no influence on the surface roughness.

The results of this study showed that air abrasion cannot be replaced by plasma treatment. Neither the investigated cleaning suspensions nor plasma treatment resulted in sufficient cleaning of CoCr surfaces contaminated with saliva and a silicone disclosing agent. Thus, plasma treatment without air abrasion cannot be recommended as the sole pretreatment in adhesive dentistry.

## Conclusions

After contamination of air-abraded CoCr alloy with saliva and silicone disclosing agent, none of the investigated cleaning methods were sufficient to produced long-term durable resin-bonding. The treatment of bonding surfaces with air plasma had no significant effect on the durability of resin bonding to CoCr alloy.

## Clinical relevance:

Air abrasion of the inner surfaces of dental alloys and zirconia ceramics with alumina particles is well established and still can be considered the gold standard for surface conditioning prior to adhesive bonding, as confirmed by the results of the current study.
